# Copper-based metal–organic frameworks: applications and prospects in cancer diagnosis and therapy

**DOI:** 10.1039/d6ra03221d

**Published:** 2026-07-03

**Authors:** Yan Huang, Junyu Zhu, Aimin Guan, Weixia Qing

**Affiliations:** a School of Basic Medical Science, Henan University Kaifeng 475004 P. R. China qingweixia@henu.edu.cn; b School of Pharmacy, Henan University Kaifeng 475004 P. R. China aiminguan@henu.edu.cn

## Abstract

Metal–organic frameworks (MOFs) have emerged as versatile nanoplatforms for biomedical applications, attributed to their architecturally tunable structures and multifunctional capabilities. Among these, copper-based MOFs (Cu-MOFs) have garnered substantial attention in oncological research due to their intrinsic physicochemical properties, including catalytic activity, photothermal responsiveness, and biocompatibility. Research has demonstrated that Cu-MOFs can effectively circumvent critical limitations of conventional therapeutic modalities, specifically chemotherapy-induced drug resistance, systemic toxicity, and insufficient tumour targeting. Through rational structural engineering, researchers have developed multimodal combination therapy systems that offer enhanced efficacy and reduced adverse effects. Recent advances have further highlighted the synergistic potential of Cu-MOFs in inducing cuproptosis and augmenting immunotherapeutic approaches. This review systematically examines recent progress (2020–2026) in the application of Cu-MOFs for cancer diagnosis and therapy, elucidates the mechanisms by which they enhance conventional treatments, analyses established combination therapy systems, and evaluates advancements in cuproptosis-related and immunotherapeutic strategies. Finally, the review discusses critical challenges in clinical translation and proposes future research directions.

## Abstract 2

This review summarizes recent advances of Cu-MOFs for cancer diagnosis and multimodal therapy, including biosensing, chemo/CDT/PDT/PTT, cuproptosis, and immunotherapy, focusing on biosafety and clinical translation challenges.

## Graphical abstract

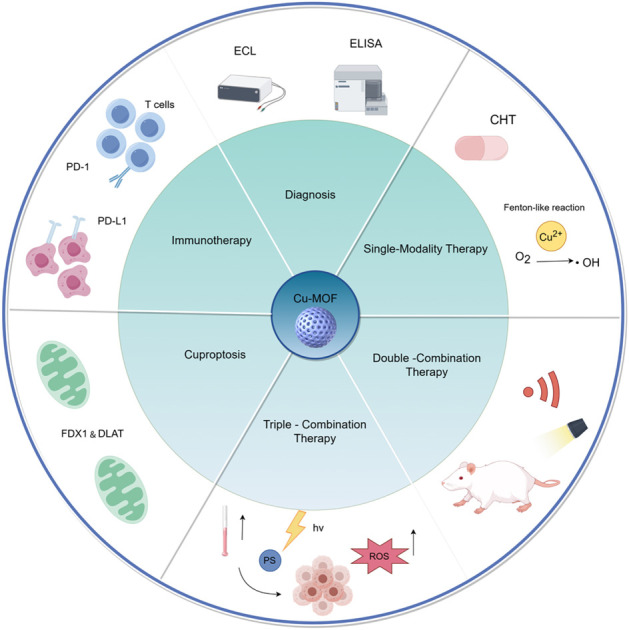

## 1 Introduction

Cancer represents a formidable global health challenge, with incidence and mortality rates escalating due to population growth and demographic aging.^1^ Current diagnostic and therapeutic paradigms confront substantial limitations: conventional detection methodologies exhibit insufficient sensitivity and procedural complexity, impeding early-stage identification;^2,3^ chemotherapeutic agents demonstrate poor target specificity, causing systemic toxicity and acquired drug resistance; radiotherapy is constrained by suboptimal tumour selectivity and limited penetration depth, particularly for anatomically inaccessible malignancies. These intertwined challenges highlight the urgent need for innovative strategies for early cancer detection and precision therapeutics. Given their unique structural and functional merits, copper-based MOFs have become a rapidly growing focus in cancer theranostics, with comprehensive reviews summarizing their diagnostic and therapeutic advances.^4^

Metal–organic frameworks (MOFs), crystalline coordination polymers composed of metal ions and organic linkers, have emerged as promising nanoplatforms for biomedical applications owing to their architecturally tunable structures and multifunctional properties.^5^ The strategic coordination of metal nodes with bridging ligands confers exceptional structural diversity and chemical programmability.^6^ Notably, MOFs possess ultrahigh surface areas (exceeding 7000 m^2^ g^−1^) and porosities (up to 94%),^7^ properties that have been extensively exploited for gas storage and separation,^8^ and chemical sensing.^9–11^ In the biomedical domain, their well-defined chemical compositions, biocompatibility, and stimuli-responsive degradability render them highly promising for controlled drug delivery^12–14^ and photothermal therapy,^15^ thereby addressing critical limitations of conventional oncological interventions.

Among various metal centres employed in MOFs, copper has attracted exceptional attention for cancer theranostics. Copper, an essential trace element in human physiology,^16^ exists in two redox states (Cu^2+^/Cu^+^) that participate in catalytic redox cycling, regulating enzymatic activity and cellular metabolism.^17,18^ Malignant cells exhibit dysregulated metal ion homeostasis during immune evasion, rendering them selectively vulnerable to copper-mediated perturbations. Within the tumour microenvironment (TME), copper exerts therapeutic effects through multiple interconnected mechanisms: (i) Cu^2+^ accumulation depletes intracellular glutathione (GSH), compromising antioxidant defences systems; (ii) Cu^+^ generates cytotoxic reactive oxygen species (ROS) *via* Fenton-like reactions;^19^ and (iii) Cu^+^ induces cuproptosis (a recently characterized form of regulated cell death), triggering immunogenic cell death (ICD) to suppress metastasis and recurrence.

**Fig. 1:**
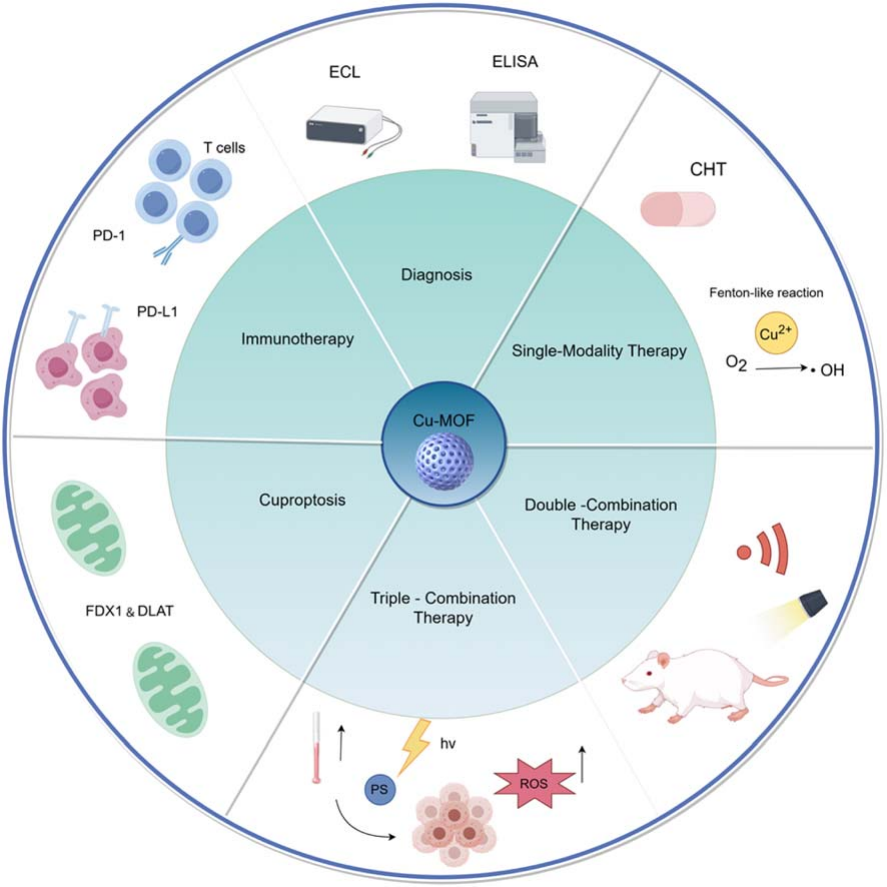
Overview of Cu-MOFs applications in cancer: covering diagnosis (ECL, ELISA), single/combination therapies, cuproptosis, and immunotherapy for multi-modal anti-tumour strategies. Image produced with Figdraw.

These distinctive physicochemical and biological attributes position copper-based MOFs (Cu-MOFs) as promising multifunctional platforms for cancer theranostics. Through rational structural engineering, Cu-MOFs enable sensitive biomarker detection for early-stage diagnosis while simultaneously circumventing the limitations of conventional therapeutic modalities.^20^ Specifically, Cu-MOFs facilitate targeted chemotherapeutic delivery to minimize off-target toxicity, enhance photothermal conversion efficiency to improve therapeutic outcomes, and modulate the tumour immune microenvironment through cuproptosis induction. Although several reviews have outlined the applications of Cu-MOFs in cancer diagnosis and therapy, most lack systematic summarization of structure–performance relationships, multimodal synergy mechanisms, and clinical translation-oriented challenges. To fill this gap, this review systematically examines recent advances (2020–2026) in Cu-MOF-based cancer management, with particular emphasis on: (i) early detection strategies leveraging Cu-MOF-enabled biosensing; (ii) mechanism-driven therapeutic optimization; (iii) multimodal combination therapy systems; and (iv) emerging applications in cuproptosis induction and immunotherapeutic synergy. Notably, recent comprehensive reviews have also validated the considerable clinical translational potential of copper-based MOFs for antitumor therapy, further motivating this indepth summarization and perspective.^21^ Finally, critical challenges in clinical translation including biosafety evaluation, scalable manufacturing, and regulatory approval are analysed, and strategic research priorities are proposed to accelerate the clinical implementation of Cu-MOF-based oncological nanomedicine. A comprehensive schematic overview of Cu-MOF applications in cancer diagnosis and therapy is presented in Fig. 1.

## 2 Cu-MOFs in cancer diagnosis

### 2.1 Biosensing platforms for tumour marker detection

Detection of tumour markers provides a critical strategy for early and precise cancer diagnosis. Common detection methods include electrochemical detection, enzyme-linked immunosorbent assay (ELISA), chemiluminescence immunoassay, electrochemiluminescence (ECL) and other types of immunoassays.^22–27^ Among various biosensing approaches, electrochemical sensing based on Cu-MOFs has been the most extensively investigated.

### 2.2 Electrochemical sensing

Cu-MOFs have been widely used in electrochemical sensing systems.^28,29^ Conventional sensors often suffer from low sensitivity, poor stability, and complex synthesis procedures. Cu-MOFs can stabilize nanoparticles, improve catalytic activity, amplify detection signals, and enhance sensing sensitivity. Their enzyme-mimetic activity and tunable structures help reduce charge interference and accelerate electron transfer, enabling highly sensitive and selective detection.^30,31^ Cu-MOFs also exhibit considerable potential to replace horseradish peroxidase (HRP) for enhanced ELISA performance.

### 2.3 Recent advances in Cu-MOF-based biosensing

**Fig. 2:**
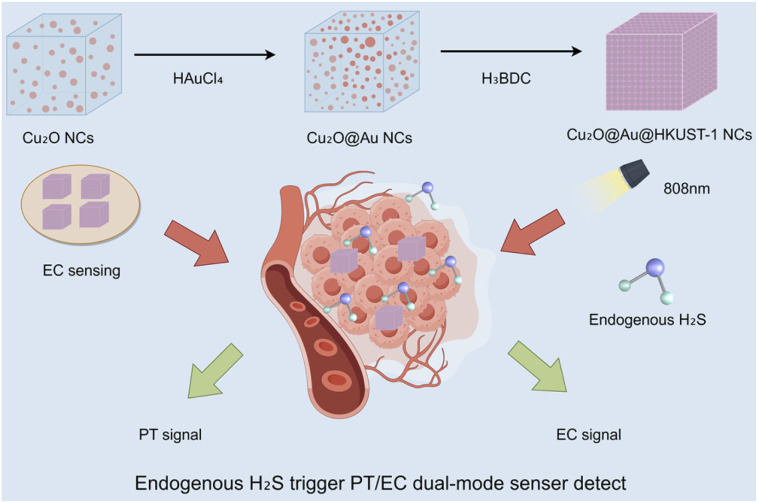
Schematic illustration of the construction of the Cu_2_O/Au/HKUST-1 nanoprobe and the endogenous H_2_S-triggered electrochemical quantification and photothermal imaging dual-channel detection of living tumour cells. Image generated with Figdraw.

Cu-MOFs have been broadly explored for sensing typical tumour markers, including microRNA,^32^ carbohydrate antigen 15-3 (CA15-3),^33^ carbohydrate antigen 19-9 (CA19-9),^34^ epidermal growth factor receptor (EGFR),^35^ human epidermal growth factor receptor 2 (HER2),^36,37^ alpha-fetoprotein (AFP),^38,39^ tumour necrosis factor-α (TNF-α),^40^ hydrogen sulfide (H_2_S) (Fig. 2),^41^ kirsten rat sarcoma viral oncogene homolog (KRAS),^42^ and hydrogen peroxide (H_2_O_2_).^43^

In recent years, various Cu-MOF-based biosensors have been developed with high sensitivity, wide linear ranges, and low detection limits. Key advances involve electrochemical, ECL, photothermal, colorimetric, and dual-modal detection strategies.^44–55^ However, most studies remain at the laboratory stage and lack validation in real clinical samples. Representative Cu-MOF-based platforms for cancer diagnosis are summarized in Table 1.

**Table 1:** Representative Cu-MOFs-based biosensing platforms for cancer diagnosis

Cu-MOFs	Analyte	LOD	Method	Ref.
Cu-NMOF@PtNPs/HRP	microRNA-155	0.13 fM	Electrochemistry	44
GO_*x*_-Cu-MOF	CA15-3	19.34 µU mL^−1^	Electro-immuno	45
Cu_*x*_Ni_3−*x*_(HHTP)_2_	EGFR	0.72 fg mL^−1^	Electrochemistry	46
Ni/Cu-MOFs	TNF-α	2.0 fg mL^−1^	Electro-immuno	47
Cu_2_O/Au/HKUST-1	H_2_S	0.1 µM	Electrochemistry	48
Cu-BTC@N-GR@AuNPs@CS	CA19-9	4.2 µU mL^−1^	Electro-immuno	49
Cu-MOF/BiVO_4_	KRAS	—	ECL	50
NG/CuMnCoO_*x*_-MOF	HER2-ECD	0.757 pg mL^−1^	Electro-immuno	51
Cu-MOFs@AuPtNPs	AFP	0.86 pg mL^−1^	ELISA	23
Au-CNMOF	H_2_O_2_	—	Electrochemistry	52
MOF@FeTCPP	HER2	28.5 fg mL^−1^	Electrochemistry	53
Cu-MOFs/SO_*x*_	CEA	43.65 fg mL^−1^	ECL	54
(Fe, Cu)-MOF-919	β-gal	—	EIS	55

### 2.4 Emerging trends and challenges

Although remarkable progress has been achieved, several critical challenges still hinder the clinical translation of Cu-MOF biosensors. Current Cu-MOF biosensors are rapidly developing toward dual-modal detection and self-calibration. However, most systems lack clinical verification in real blood or tissue samples. Long-term stability, batch reproducibility, scalable fabrication, and cost-effectiveness for point-of-care testing remain major challenges. Future research should focus on clinical translation and integrated diagnostic device development.

## 3 Cu-MOFs in cancer therapy

### 3.1 Single-modality therapy

#### 3.1.1 Chemotherapy (CHT)

Chemotherapy remains a cornerstone of cancer treatment but often damages normal cells.^56,57^ Cu-MOFs are promising carriers with high drug-loading, biodegradability, and targeted delivery, thus reducing side effects.^58–60^

Alginate-LDH-Cu-MOF showed pH-responsive release with doxorubicin (DOX) encapsulation up to 97.5%.^61^ In anticancer drug research, disulfiram (DSF) has shown considerable therapeutic potential.^62^ Studies have indicated that nanocarriers such as Cu-MOF enhance its therapeutic efficacy.^63,64^ Co-delivery and localized release of DSF with copper at the tumour site can significantly improve chemotherapy outcomes. Hyaluronic acid (HA), which exhibits strong affinity for the cluster of differentiation 44 (CD44) receptor overexpressed on various tumour cell surfaces, can be used to functionalize nanocarriers and enhance tumour targeting.^65–67^ Leveraging this property effectively boosts the targeting ability of Cu-MOFs in drug delivery. Li and colleagues synthesized Cu-BTC@DDTC, in which the complexation of copper ions with diethyldithiocarbamate (DDTC), a serum metabolite of DSF, activates anticancer activity.^68^ This copper-dependent killing is mediated by FDX1-driven cuproptosis rather than classical apoptosis: FDX1 transports Cu^+^ into mitochondria to disrupt the TCA cycle and induce lipoylated protein aggregation, which is fundamentally different from the DNA damage pathway triggered by traditional chemotherapeutics. The Cu-MOF increased intracellular copper ion concentration, thereby enhancing DSF's therapeutic effect on tumours. The Cu-BTC@DDTC system exhibited high drug-loading capacity and strong stability. Compared with the control group, the Cu-BTC@DDTC group significantly promoted tumour cell apoptosis at a dose of 20 mg kg^−1^.

Recent advances have further expanded the chemotherapeutic applications of Cu-MOFs. In 2025, a flexible supramolecular metal–organic framework (Cu_7_Naph) was developed as a multifunctional carrier for the co-delivery of cisplatin and 5-fluorouracil, enabling synergistic anticancer effects with synchronized release kinetics.^69^ Additionally, a clinically inspired olsalazine-based Cu/Fe MOF was designed to encapsulate DOX while addressing endogenous H_2_O_2_ deficiency in the TME through a self-catalytic H_2_O_2_ regeneration mechanism.^70^ These innovations highlight the growing versatility of Cu-MOFs in combination chemotherapy strategies.

##### 3.1.1.1 Critical assessment

Although Cu-MOF-based chemotherapy demonstrates promising preclinical efficacy, critical gaps persist. The majority of studies have focused on short-term cytotoxicity assessments *in vitro*, with limited long-term *in vivo* efficacy and toxicity profiling. Furthermore, the pharmacokinetic behaviour, biodistribution, and clearance pathways of Cu-MOFs in animal models remain incompletely characterized. The potential for copper-induced systemic toxicity, particularly hepatic and renal accumulation, warrants comprehensive investigation. Clinical translation will require rigorous Good Manufacturing Practice (GMP)-compliant synthesis protocols and standardized quality control metrics.

#### 3.1.2 Chemodynamic therapy (CDT)

CDT exerts antitumor effects by converting endogenous H_2_O_2_ into cytotoxic ˙OH *via* Fenton or Fenton-like reactions, inducing lipid peroxidation, protein inactivation, and DNA damage.^71,72^ However, CDT efficiency is severely restricted by high glutathione (GSH) levels, non-optimal pH, and insufficient H_2_O_2_ in the TME. Cu-MOFs address these bottlenecks through Cu^2+^/Cu^+^ redox cycling that depletes GSH, catalyses Fenton-like reactions over a wide pH range, and enhances H_2_O_2_ generation when loaded with glucose oxidase (GOD).^73–77^

In 2022, Xu and colleagues designed a nanotherapeutic platform encapsulating Cu-T *n*MOF with MnO_2_.^78^ The acidic TME and elevated H_2_O_2_ levels trigger the release of Cu-T *n*MOF, promoting ROS generation and GSH depletion to enhance CDT efficacy. Fluorescence recovery of TCPP in Cu-T *n*MOF enables real-time therapeutic monitoring. Histological analysis *via* H&E staining revealed nuclear dissolution in tumour tissues, confirming potent antitumor activity. In 2024, Bai and colleagues synthesized two-dimensional AuTPyP-Cu nanosheets by assembling AuTPyP and Cu^2+^.^79^ Stimulation by the TME induces ferredoxin-1 (FDX1)-mediated conversion of Cu^2+^ to Cu^+^, activating cuproptosis and generating ˙OH *via* H_2_O_2_ cascade reactions. Notably, cuproptosis is a unique form of regulated cell death that is mechanistically distinct from apoptosis, ferroptosis, pyroptosis, and necroptosis. FDX1 acts as the central regulator by reducing Cu^2+^ to Cu^+^ and targeting copper ions into the mitochondrial matrix. Inside mitochondria, Cu^+^ directly binds to lipoylated proteins in the tricarboxylic acid (TCA) cycle, triggering protein aggregation, iron-sulfur cluster collapse, and proteotoxic stress, rather than caspase activation, lipid peroxidation, or membrane rupture. This strict mitochondrial metabolism-dependent mechanism enables Cu-MOFs to overcome drug resistance in apoptosis- or ferroptosis-insensitive tumour cells.

Au(iii) in AuTPyP binds to overexpressed thioredoxin reductase (TrxR) in tumours, thereby increasing ROS production. This synergistic mechanism achieved an inhibition rate of 81.3% in HeLa cells, highlighting the improved efficacy of CDT. Ren and colleagues constructed HKUST@CA by loading cinnamaldehyde (CA) onto Cu-MOF, followed by modifications with triphenylphosphine (TPP) and polyethylene glycol (PEG).^80^ In mitochondria, H_2_O_2_ triggers the release of CA, which upregulates endogenous H_2_O_2_ levels for cyclic release. The continuous generation of H_2_O_2_ during this mitochondrial self-cycling produces abundant ˙OH, leading to oxidative damage and potentiating CDT. Experimental results showed less than 10% viability of HeLa cells at a concentration of 27.4 µg mL^−1^, demonstrating robust therapeutic effects.

##### 3.1.2.1 Critical assessment

While Cu-MOF-mediated CDT demonstrates promising preclinical results, several mechanistic and translational challenges require attention. First, the relative contributions of Fenton chemistry *versus* cuproptosis induction in Cu-MOF-mediated tumour cell death remain incompletely disentangled. Second, the long-term biological fate of copper ions released from Cu-MOFs, particularly regarding potential accumulation in non-target organs, necessitates comprehensive toxicological evaluation. Third, the clinical relevance of CDT is constrained by the substantial inter- and intra-tumoral heterogeneity in H_2_O_2_ and GSH levels, suggesting that patient stratification strategies may be essential for effective clinical implementation.

#### 3.1.3 Photodynamic therapy (PDT)

Photodynamic therapy (PDT) primarily employs photosensitizers (PSs) to generate singlet oxygen (^1^O_2_) under light irradiation, inducing apoptosis in target tumour cells for cancer treatment.^81,82^ However, the efficacy of PDT is often limited by multiple factors including poor PS targeting, low safety, high GSH levels, and hypoxia in the TME, which significantly hinder its effectiveness.^83–85^ Cu-MOFs, such as Cu-TCPP, can reduce intracellular GSH levels, thereby enhancing PDT efficacy.^86,87^ They can also effectively address tumour hypoxia, and their encapsulation ability mitigates PS toxicity to normal tissues.

In 2019, Lin and colleagues developed an O_2_-loaded CuTz-1@F127 MOF therapeutic system to improve PDT by alleviating hypoxia and reducing GSH levels in tumour cells.^88^ The CuTz-1 MOF acts as a PS, generating ˙OH and O_2_ in the presence of H_2_O_2_. The CuTz-1@F127 composite delivers O_2_ into cancer cells and adsorbs intracellular GSH, simultaneously alleviating hypoxia and GSH overexpression and significantly enhancing PDT efficacy. Mouse experiments showed that at a concentration of 20 mg kg^−1^, the body weights of the treated mice remained stable over time, indicating good biosafety. Wang and colleagues encapsulated PS in MOF-199 to isolate oxygen and prevent PS photosensitization.^89^ They then encapsulated PS@MOF-199 with F-127. In tumour tissues, MOF-199 is decomposed by GSH, releasing the loaded PS, which restores its photosensitivity and initiates PDT. This approach effectively reduces endogenous GSH levels, minimizes normal-tissue toxicity, and significantly enhances cancer treatment efficacy and safety. In 2022, Liu and colleagues synthesized a smart nanodrug, Cu-TCPP(Al)Pt-FA, which enhances PDT by generating O_2_*in situ* and depleting GSH.^90^ Cu^2+^ in two-dimensional MOFs can specifically bind and adsorb intracellular GSH. Leveraging the catalase-like activity of PtNPs, Cu-TCPP(Al)-PtFA continuously converts intracellular H_2_O_2_ into O_2_, alleviating tumour hypoxia.

##### 3.1.3.1 Critical assessment

Despite promising preclinical outcomes, Cu-MOF-enhanced PDT faces substantial translational hurdles. The limited tissue penetration depth of activating light (typically <1 cm for visible light) restricts applicability to superficial or endoscopically accessible tumours. Furthermore, the potential for phototoxicity in normal tissues exposed to ambient light following PS administration requires careful management. The development of Cu-MOF formulations with optimized pharmacokinetic profiles to maximize tumour-to-normal tissue PS concentration ratios remains an active area of investigation.

#### 3.1.4 Photothermal therapy (PTT)

Photothermal therapy (PTT) utilizes near-infrared (NIR) light to irradiate photothermal agents, leveraging tumour cells' heat sensitivity to induce localized hyperthermia and subsequent cell death.^91–94^ While relatively safe, PTT efficacy is limited by the shallow penetration depth of NIR light, restricting its effectiveness as a standalone treatment for deep-seated tumors.^95^ Cu-MOFs enhance the photothermal conversion efficiency of PTT agents, thereby improving therapeutic outcomes.

In 2020, Weng and colleagues developed an innovative photothermal agent, Cu@CPP-800, which demonstrated strong NIR absorption and exceptional photothermal conversion properties.^96^ Under 808 nm laser irradiation, Cu@CPP-800 achieved a photothermal conversion efficiency of up to 48.5%. Dang and colleagues synthesized Cu-TCPP nanosheets and integrated them into 3D-printed vascular scaffolds for osteosarcoma treatment.^97^ These nanosheets demonstrated a 36.8% photothermal conversion efficiency, depending on Cu-TCPP content. *In vivo* mouse experiments confirmed that the Cu-TCPP-TCP scaffold ablated subcutaneous bone tumour tissue and inhibited growth *via* NIR-induced hyperthermia. In 2023, Chung and colleagues fabricated Cu-BTC@PDA nanowires, utilizing polydopamine (PDA) to enhance NIR absorption.^98^ Cu-MOFs were chosen for their favourable photothermal properties and high-temperature responsiveness. The PDA-coated nanowires exhibited good photothermal stability, with 808 nm laser irradiation eliminating 58% of tumour cells and demonstrating superior therapeutic performance.

##### 3.1.4.1 Synthesis and critical assessment

Collectively, Cu-MOFs offer distinct advantages in single-modality therapies: enhancing targeted drug delivery in CHT, boosting Fenton-like reactions and depleting GSH in CDT, improving photosensitizer delivery in PDT, and increasing photothermal conversion efficiency in PTT. However, each modality has inherent limitations: CHT faces drug resistance and toxicity; CDT efficacy is constrained by TME conditions; PDT is hindered by hypoxia; and PTT suffers from limited light penetration. The invasive and heterogeneous nature of tumours renders single-modality approaches insufficient. Consequently, developing Cu-MOF-based combination therapies represents a strategic solution to address these shortcomings and enhance treatment efficacy. Given the inherent drawbacks of single-modal therapy, increasing efforts have been devoted to developing dual-combination therapeutic systems based on Cu-MOFs.

### 3.2 Dual-combination therapy

Dual-combination therapy achieves synergistic effects by complementary mechanisms, and the performance of different systems is highly determined by pore structure, copper redox chemistry, stimulus responsiveness, and functional integration of Cu-MOFs. Below is a comparative analysis of representative strategies.

#### 3.2.1 CHT-CDT

The CHT-CDT combination operates *via* a bidirectional amplification loop: CDT activates prodrugs carried by Cu-MOFs to enhance CHT precision, while CHT agents elevate endogenous H_2_O_2_ levels to reinforce CDT activity.

Structurally, pH/GSH-responsive Cu-MOFs (DOX-loaded core–shell Cu-MOF@SMON/DOX-HA) enable controlled release of Cu^2+^, DOX, and 3-AT. The Cu^2+^/Cu^+^ redox cycle drives Fenton-like reactions and depletes GSH efficiently, while 3-AT reduces H_2_O_2_ loss, yielding a 50% GSH reduction and favourable drug loading (27.5%).^99^ By contrast, graphene-integrated GDY-CuMOF@DOX benefits from high electronic conductivity and membrane coating (DCM), which enhance tumor targeting and stability. This system achieves 77.6% drug loading and 97% DOX encapsulation, triggering both ROS-mediated apoptosis and cuproptosis to suppress migration and invasion.^100^ Ligand-engineered systems (DCCMH) use metformin and CHCA to acidify the TME and boost chemosensitivity, while Cu^+^-catalysed ROS production reinforces antitumor action.^101^

Compared with simple Cu-MOFs, core–shell and carbon-integrated structures show stronger stability, higher loading, and better TME adaptation; however, they involve more complex synthesis. TME-responsive and hypoxia-activated Cu-MOF platforms further expand CHT-CDT applications by combining CDT with gene therapy or cuproptosis, improving compatibility and efficacy against hypoxic tumours.^102,103^

#### 3.2.2 CDT-PDT

Tumour hypoxia severely weakens PDT efficacy. CDT-PDT synergy resolves this issue: Cu^2+^-mediated CDT consumes GSH to relieve oxidative resistance, while Cu-MOFs act as carriers for PS to support PDT. Fluorescence guidance further improves targeting accuracy.

ZIF-8-based A-NUiO@DCDA@ZIF-Cu provides a typical example: the ZIF-8 shell releases Cu^2+^ to drive Fenton-like reactions, while the AIEgen-containing core enhances imaging and *in situ* O_2_ generation to relieve hypoxia. This design synchronizes imaging, CDT, and O_2_-independent PDT.^104^ HN@Cu-MOF integrates CDT, PDT, and NO therapy: under laser irradiation, Cu^2+^ depletes GSH, and NO release alleviates hypoxia, amplifying ROS-mediated tumour suppression.^105^ Structurally, ZIF-series Cu-MOFs excel in pH responsiveness and biocompatibility; AIEgen-integrated systems improve imaging and PDT stability. However, these systems rely on light excitation and remain less effective for deep-seated tumours.

#### 3.2.3 CDT-PTT

CDT-PTT synergy forms a positive-feedback loop: photothermal heating accelerates Fenton-like kinetics to increase ˙OH production, while enhanced oxidative stress sensitizes cells to thermal damage.

HKUST-1 responds to endogenous H_2_S by forming CuS *in situ*, which provides strong NIR absorption and high photothermal conversion efficiency (45.7%), enabling synergistic nanozyme-mediated CDT and PTT with >80% tumour inhibition.^106^

Cu_2−*x*_Se@ZIF-8/ICG combines Cu^+^-driven CDT with ICG-supported PTT, showing high biocompatibility and efficacy against bone metastases.^107^ ZnS/Cu_2_O@ZIF-8@PVP releases Cu^+^ in acidic TME to generate CuS photothermal agents *in situ*, allowing real-time photothermal imaging and 63.1% apoptosis induction.^108^ Au-based photothermal effects and Cu-based Fenton catalysis, reaching 57.45% photothermal conversion efficiency with reliable biosafety.^109^ Comparatively, HKUST-1 and ZIF-8-based heterostructures offer strong stimulus responsiveness and multifunctionality; Au-containing hybrid systems provide higher photothermal efficiency but require more complex fabrication.

#### 3.2.4 PDT-PTT

Photothermal effects improve PDT by relieving tumour hypoxia, allowing PDT-PTT to eradicate tumours synergistically.^110^ Cu-MOFs can act as dual-function PS carriers and phototransducers, although many require ICG to compensate for weak NIR absorption.

**Fig. 3:**
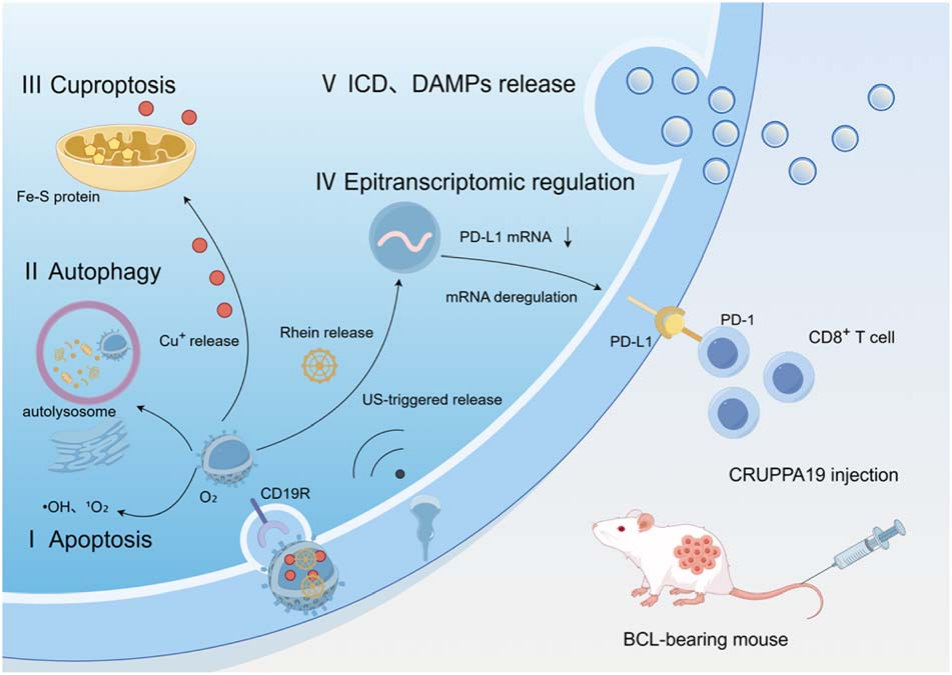
Schematic of MOF-based sono-epigenetic immunotherapy for B-cell lymphoma. Ultrasound triggers cascade apoptosis, autophagy, cuproptosis, and epitranscriptomic regulation to boost anti-tumour immunity. Image produced with Figdraw.

2D Cu-TCPP nanosheets exhibit strong light harvesting and fast charge migration, supporting balanced PDT-PTT.^111^ ICG-loaded platforms further enhance photothermal performance and enable dual-mode imaging guidance, achieving robust tumour growth suppression.^112^ For deep-seated tumours, sonodynamic-immunotherapy (SDT-based therapy) is more favourable due to deep ultrasound penetration.^113–115^ UiO-66-NH_2_-supported CuR@UiO66 (CRUPPA19) provides ultrahigh CuRhein loading (98%) and CD19 targeting for B-cell lymphoma.^116^ Under US irradiation, this system triggers ROS generation, apoptosis, cuproptosis, and PD-L1 downregulation, promoting immunogenic cell death (ICD) and CD8^+^ T-cell priming. It achieves 94.3% lymphocyte killing *in vitro* and 90.4% distant tumor inhibition *in vivo*, outperforming light-triggered platforms for deep and metastatic tumours (Fig. 3).

To further amplify therapeutic efficacy and overcome drug resistance, triple-combination therapy based on Cu-MOFs has emerged as a more powerful strategy.

### 3.3 Triple-combination therapy

Triple-mode combination therapy further enhances antitumour efficacy *via* multi-mechanism synergy, reduced dosages, and reversed drug resistance. These systems impose stricter requirements on structural stability, multi-component loading, sequential responsiveness, and biodegradability of Cu-MOFs.

#### 3.3.1 CHT-CDT-PDT

This strategy integrates chemotherapy, oxidative damage, and hypoxia relief.^117–119^ Cu@MIL-101-based platforms enable co-loading of chemotherapeutics, photosensitizers, and immunomodulators, with ordered pores that ensure stable release and low systemic toxicity.^120^ Cu^2+^-catalysed CDT and laser-activated PDT amplify ROS levels to sensitize cells to DOX, yielding tumour inhibition rates exceeding 89%. Hierarchical porosity improves mass transport but may reduce structural robustness. The multi-component design delivers strong synergy but complicates batch consistency and clinical translation.

#### 3.3.2 CHT-CDT-PTT

Thermal activation drives synchronized drug release and Fenton enhancement. ZIF-8@PDA core–shell structures combine sharp pH responsiveness, stable photothermal performance, and controlled drug release.^121^ Hyperthermia accelerates both Cu^+^-mediated ˙OH production and DOX release, creating a one-trigger multi-effect system. Highly crystalline Cu-MOFs support durable catalytic activity but show slower degradation and less controllable Cu^2+^ release. Challenges include non-uniform intratumoral heating and limited scalability.

#### 3.3.3 CDT-PDT-PTT

**Fig. 4:**
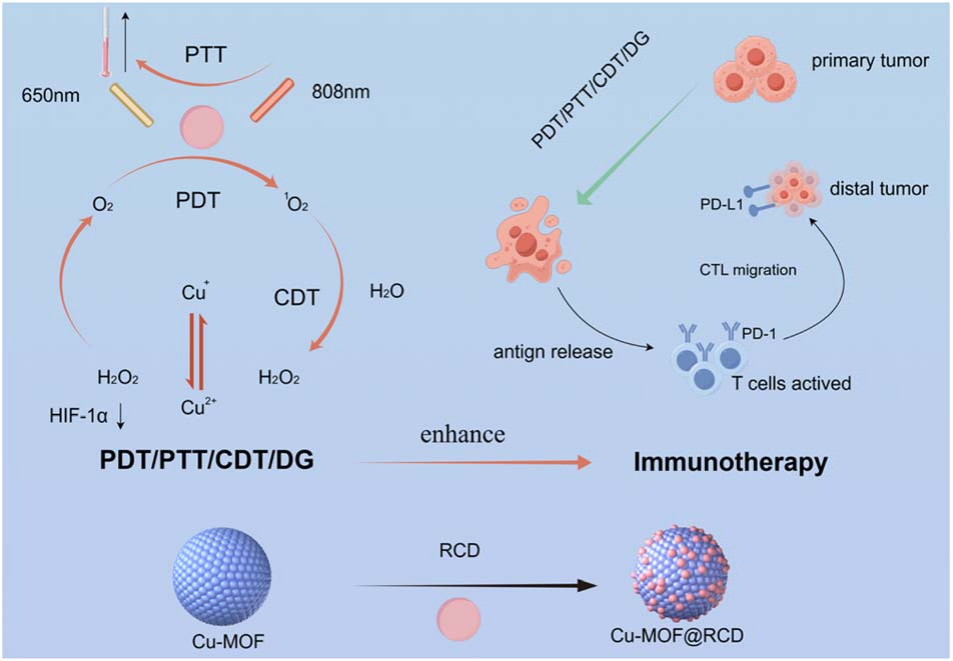
Synthesis of Cu-MOF@RCD and its mechanism: PDT/PTT/CDT/DG synergistically trigger immunogenic cell death, activating T cells to inhibit both primary and distant tumour. Image produced with Figdraw.

This chemo-free therapeutic regimen achieves potent anti-tumour effects through synergistic oxidative stress and photothermal ablation. In 2023, Jin and co-workers developed CuMOF@RCD as an integrated nanoreactor for CDT-PDT-PTT.^122^ Upon 650 nm laser irradiation, RCD mediated PDT to generate ROS and oxygen, relieving hypoxia-induced PDT resistance. Under 808 nm irradiation, the photothermal effect raises local temperature to accelerate Fenton-like reactions and enhance therapeutic outcomes. This system achieves a photothermal conversion efficiency of 32.7% and a 97.8% primary tumour growth inhibition rate under dual-wavelength activation (Fig. 4). In 2024, Yuan and colleagues constructed PCN-224(Cu)@PDA (PCP) nanoparticles embedded in oxidized sodium alginate and carboxymethyl chitosan.^123^ Under 660 nm and 808 nm NIR light, the porphyrin backbone supports PDT while the composite structure enables PTT. Following intratumoral injection, PCP is efficiently internalized by tumour cells to realize precise, synergistic CDT-PDT-PTT therapy.

Before clinical translation can be realized, the biosafety, biodegradation, and long-term biocompatibility of Cu-MOFs must be systematically evaluated.

### 3.4 Biosafety and clinical translation of Cu-MOFs

Successful clinical translation of Cu-MOFs relies on systematic evaluations of biosafety, biodistribution, biodegradation, metabolic clearance, and long-term biocompatibility.^124,125^ The therapeutic efficiency of Cu-MOFs is highly susceptible to the heterogeneity of the TME. Acidic pH activates framework degradation, high GSH accelerates Cu^2+^ reduction to Cu^+^ and promotes cuproptosis, insufficient H_2_O_2_ limits CDT efficacy, and hypoxia suppresses PDT/SDT and upregulates copper efflux proteins, collectively reducing therapeutic stability. Excess released copper ions disrupt redox homeostasis, induce oxidative stress, and impair normal organs, especially the liver and kidneys, which are major accumulation sites. Systemically administered Cu-MOFs mainly accumulate in the reticuloendothelial system and degrade in the tumour microenvironment, releasing copper ions and ligands gradually. Metabolites are excreted *via* renal and hepatobiliary pathways, while incomplete degradation may cause long-term retention and inflammation. Long-term risks such as cumulative organ toxicity and potential immunogenicity remain to be fully elucidated. Rational structural design and standardized GMP synthesis are critical to enhance biocompatibility and ensure clinical safety.

Critical evaluation of targeting strategies: hyaluronic acid (HA) modification offers high targeting efficiency and easy synthesis but suffers from off-target binding and low specificity. Membrane coating provides excellent biocompatibility and immune escape but has poor batch reproducibility and low loading stability. Antibody modification achieves precise targeting but increases immunogenicity, raises production cost, and causes structural instability during storage.

Further efforts toward clinical translation should focus on developing scalable and green synthetic routes, improving batch-to-batch reproducibility, establishing strict GMP-compliant manufacturing protocols, enhancing physiological and structural stability, and comprehensively addressing regulatory requirements, safety assessments, and quality control standards.

## 4 Cu-MOFs in cuproptosis and immunotherapy

Cuproptosis, a novel form of programmed cell death discovered by Tsvetkov and colleagues in 2022,^126^ is characterized by excessive intracellular accumulation of Cu^2+^. These ions directly interact with lipoylated proteins in the mitochondrial tricarboxylic acid (TCA) cycle, triggering lipoylated protein aggregation, loss of iron-sulfur clusters, and upregulation of heat shock protein 70 (HSP70). This cascade induces proteotoxic stress and ultimately cell death.^127,128^ Cancer immunotherapy utilizes the immune system to recognize and eliminate cancer cells *via* immunogenic cell death (ICD), induced by chemotherapy, radiotherapy, or photodynamic therapy. These treatments induce cellular stress, generate ROS, disrupt endoplasmic reticulum (ER) structure and function, and promote release of damage-associated molecular patterns (DAMPs), including calreticulin (CRT), high-mobility group box-1 protein (HMGB1), and adenosine triphosphate (ATP).^129–131^ These molecular signals drive dendritic cell (DC) maturation, recruit cytotoxic T lymphocytes (CTLs) to tumour sites, and eliminate cancer cells.^132–135^ ICD also fosters immune memory, inhibiting metastasis, recurrence, and drug resistance.^136,137^ The activation of regulated cell death (RCD) can potentiate antitumor immunogenicity and enhance cancer immunotherapy.^138^ As a novel RCD modality, cuproptosis shows promise in immunology and oncology.^139,140^ Mitochondrial copper accumulation activates macrophages into an inflammatory M1-like phenotype, boosting antitumor immunity.^141,142^ Additionally, copper modulates PD-L1 expression, reversing immunosuppressive TMEs and improving immunotherapy efficacy.

However, non-selective copper accumulation causes systemic toxicity and damages normal tissues.^143^ Copper ionophores such as elesclomol (ES) facilitate FDX1-dependent transport of extracellular Cu^2+^ into mitochondria, inducing cuproptosis selectively in tumour cells while sparing normal cells.^144,145^ Cu-MOFs, emerging as ideal copper carriers due to high loading capacity, stability, and biocompatibility, induce cuproptosis and enhance immunotherapy.

**Fig. 5:**
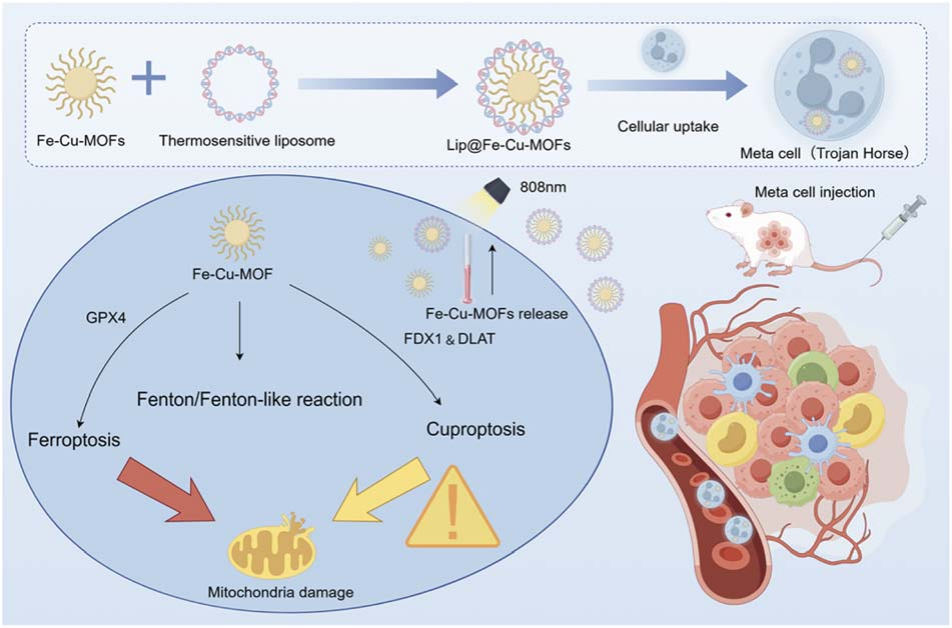
Schematic of MetaCell Trojan Horse fabrication and mechanism. Lip@Fe-Cu-MOFs are taken up to form MetaCell, which release Fe-Cu-MOFs under 808 nm light, triggering synergistic cuproptosis and ferroptosis for tumour therapy. Image produced with Figdraw.

In 2024, Lu and colleagues synthesized ES@Cu-MOF, a Cu-MOF nanocomposite loaded with ES.^146^ This platform activated cuproptosis, exhibited enzyme-like activity, generated ˙OH radicals, and depleted GSH. Experiments in 4T1 mouse model showed that ES@Cu-MOF-induced cuproptosis significantly inhibited tumour growth, with ICD-triggered synergistic suppression achieving over 85% tumour growth inhibition. Luo and colleagues developed ES-Cu-MOF, a targeted nanoplatform combining ES and Cu-MOF for cancer immunotherapy.^147^ It enabled targeted release of Cu^2+^ and ES, inducing cuproptosis and ICD *via* FDX1 depletion and mitochondrial ROS elevation. Intravenous injection led to tumour accumulation, DC activation, and fibrosarcoma growth inhibition. Zhao and colleagues designed OMP, a copper-containing MOF system loaded with PDK1-targeting siRNA for inhaled lung metastasis treatment.^148^ pH-responsive siPDK release downregulated PDK1, inhibiting glycolysis and ATP production. OMP-induced cuproptosis triggered ICD, enhanced antitumor immunity, and upregulated membrane-associated PD-L1 (mPD-L1), synergizing with PD-L1 checkpoint blockade. Shi and colleagues fabricated PCu-HA-DQ by loading DQ onto PCu-MOFs and coating with HA.^149^ In the TME, H_2_O_2_ triggered ^1^O_2_ generation for ICD, while DQ released diethyldithiocarbamate (DTC) to chelate Cu(ii), enhancing DSF effects. This combined CHT, CDT, and anti-PD-1 immunotherapy to reshape the immune microenvironment. Chen and colleagues developed a GSH-responsive CaCu-based MOF loaded with DOX and ovalbumin (OVA) for multimodal therapy.^150^ SCC/DOX@OVA-HG degraded in GSH-rich tumours, releasing Cu^+^ for CDT/cuproptosis and Ca^2+^ for calcium-mediated cell death. OVA and metal ions activated macrophages, enhancing immunotherapy. Jiang and colleagues designed Cu_2−*x*_Se@cMOF for sonodynamic cuproptosis/gas combination therapy,^151^ regulating Cu^2+^/Cu^+^, H_2_O_2_, and GSH. Elevated copper upregulated PD-L1, enhancing anti-PD-L1 efficacy and driving DC maturation, CD8^+^ T cell infiltration, and M2-to-M1 macrophage polarization. Huang and colleagues combined prodigiosin with Cu-based nanomaterials to create ZCProP-NPs,^152^ inducing mitochondrial dysfunction and DNA damage *via* mitochondria-targeted, ATP/pH-responsive Cu^2+^ release. GSH depletion promoted cuproptosis and ferroptosis, demonstrating potent, biocompatible antitumor effects. Ning and colleagues engineered Metacell by loading Fe–Cu bimetallic MOF into thermosensitive liposomes within neutrophils.^153^ Neutrophil chemotaxis targeted tumours, inducing cuproptosis and ferroptosis exacerbated by local heating. Fluorescence imaging showed photothermal effects increased tumour inflammation, attracting more Metacell (Fig. 5). In 2025, Xie and colleagues designed CaCuZC for combined cuproptosis-ferroptosis and calcium overload immunotherapy.^154^ In acidic, GSH-rich environments, CaCuZC released Cu^2+^, IR780CD, and CaO_2_, generating Ca^2+^/H_2_O_2_. Cu^2+^ induced cuproptosis, while Ca^2+^ overload caused mitochondrial damage and ferroptosis *via* GPX4 inactivation and lipid peroxidation. Released DAMPs activated antitumor immunity.

Critical assessment and mechanistic insights: the therapeutic potential of cuproptosis hinges on tumour cells' abnormal copper metabolism and their reliance on TCA cycle signaling.^155–162^ Cu-MOFs, as efficient copper ionophores, induce cuproptosis and ICD, enhancing immunotherapy with strong tumour targeting, low normal tissue toxicity, and circumvention of chemo- and radiotherapy limitations such as resistance and side effects. Representative Cu-MOFs-based nanoplatforms for cancer therapy are summarized in Table 2.

**Table 2:** Representative Cu-MOF-based nanoplatforms for cancer therapy

Cu-MOFs system	Modification/loaded components	Therapeutic modalities	Ref.
**Single-modality**			
Alg-DOX-Cu-MOF-LDH	DOX	CHT	61
Cu-BTC@DDTC	DDTC	CHT	68
2DCu-T *n*MOFs	MnO_2_	CDT	78
GO_*x*_@Cu-ZIF8-3AT@HA	HA	CDT	77
2D AuTPyP-CuS	—	CDT	79
CuTz-1O_2_	F127	PDT	88
Cu-TCPP-(Al)-Pt-FA	Pt NPs/HA	PDT	90
Cu@CPP-800	—	PTT	96
Cu-TCPP-TCP	TCP	PTT	97
Cu-BTC@PDA NWs	PDA	PTT	98
**Dual-modality**			
Cu-MOF@SMON/DOX-HA	SMON/HA/DOX	CHT-CDT	99
DCM@GDY-CuMOF@DOX	DCM/DOX	CHT-CDT	101
A-NUiO@DCDA@ZIF-Cu	DCDA	CDT-PDT	104
HN@Cu-MOF	HA	CDT-PDT	105
ICG@Cu_2−*x*_Se-ZIF-8	ICG	CDT-PTT	107
ZnS/Cu_2_O@ZIF-8@PVP	PVP	CDT-PTT	108
Au@Cu_5_Zn_8_/HPCNC	AuNPs	CDT-PTT	109
2D Cu-TCPP	—	PDT-PTT	111
PCN-CuS-FA-ICG	ICG/PVP/HA	PDT-PTT	112
CuR@UiO66	—	SDT-CDT	116
**Triple-modality**			
MOF(Cu)@Dox-PL NPs	DOX	CHT-CDT-PDT	117
Cu@MIL-101@PMTPC	Pt/1 MT/TCPP	CHT-CDT-PDT	118
HCPT@Cu/ZIF@PDA	HCPT/PDA	CHT-CDT-PTT	121
Cu-MOF@RCD	RCD	CDT-PDT-PTT	122
PCN-224(Cu)@PDA	PDA	CDT-PDT-PTT	123
**Immuno-combination**			
ES@Cu(ii)-MOF	ES	Cuproptosis-immuno	146
PCu-HA-DQ	DSF/HA	CHT-CDT-immuno	149
Cu_2−*x*_Se@cMOF	—	SDT-cuproptosis-immuno	151
IF90/Cu-prodigiosin@PEG	PEG/prodigiosin	Cuproptosis-ferroptosis	152
Fe-Cu-MOFs	Liposome	Cuproptosis-ferroptosis	153
CaO_2_@CuZIF/CD	HA	CDT-SD-cuproptosis-ferroptosis	154
Cu-MOF	DPCPX	Cuproptosis-ferroptosis-mitophagy	163
FA-PZ@MOF	FA	Cuproptosis-pyroptosis-immuno	164
Fe-Cu-MOF	MnO_2−*x*_	Cuproptosis-sono-immuno	165
ZCESM@mem	ES	Cuproptosis-PTT-immuno	166
CMC	Cu_2_O	Cuproptosis-sono-immuno	167

Recent advances have further expanded the therapeutic landscape of Cu-MOFs. A pH-responsive Cu-MOF nanoplatform (Cu-MOF@DPCPX) was engineered to co-trigger cuproptosis, ferroptosis, and mitophagy through tumour-specific copper overload, demonstrating potent tumour suppression across multiple tumour models and reprogramming the immunosuppressive TME *via* increased CD8^+^ T-cell infiltration and M1 macrophage polarization.^163^ Another study developed tumour-targeting Cu-MOF nanoparticles that simultaneously induce cuproptosis and pyroptosis in hepatocellular carcinoma cells, triggering robust ICD while inhibiting tumour metastasis and invasion.^164^ A bimetallic Cu/Fe-MOF-based heterojunction sonozyme was recently reported for triple amplification of sono-immunotherapy through activating tumour-specific cuproptosis and the cGAS-STING pathway, achieving complete elimination of primary tumours and significant control of distant tumour growth.^165^ Additionally, a biomimetic nanoplatform was designed to achieve efficient copper ion transport for the combination of cuproptosis and *in situ* PTT, evoking robust immune responses to inhibit tumour proliferation and recurrence.^166^ An *in situ* heterojunction strategy using a Cu-MOF protective layer to regulate the biodegradation of copper-based nanomaterials has also been developed for tumour-specific cuproptosis-enhanced sono-immunotherapy.^167^ These recent studies highlight the rapidly evolving frontier of Cu-MOFs in integrating multiple cell death pathways and immune activation mechanisms for enhanced cancer therapy.

In summary, the unique structural and biological merits endow Cu-MOFs with unparalleled advantages in cancer theranostics.

## 5 Conclusions and prospects

Cu-MOFs have emerged as versatile theranostic platforms enabling high-sensitivity tumour biomarker detection and multimodal synergistic therapy, effectively addressing limitations of conventional oncological interventions. Cuproptosis induction further empowers Cu-MOFs to trigger ICD and reshape the immune microenvironment, offering new avenues to overcome drug resistance and inhibit metastasis.

Key future directions should focus on: (i) establishing scalable, GMP-compliant synthesis with rigorous quality control; (ii) conducting long-term biosafety and pharmacokinetic evaluations to clarify copper ion fate *in vivo*; (iii) dissecting crosstalk between cuproptosis, ferroptosis, apoptosis and ICD *via* genetic and pharmacological tools; (iv) developing predictive biomarkers for patient stratification toward personalized therapy; (v) optimizing dual-mode sensing and deep-tissue treatment modalities to enhance clinical applicability; (vi) engaging regulatory authorities early to streamline translation of Cu-MOF-based nanomedicines. With systematic resolution of synthesis, safety and mechanistic challenges, Cu-MOFs are poised to advance precision cancer theranostics and bring substantial clinical benefits to patients.

## Conflicts of interest

There are no conflicts to declare.

## Data Availability

No primary research results, software or code have been included and no new data were generated or analysed as part of this review.
